# Genetic Characterization of Middle East Respiratory Syndrome Coronavirus, South Korea, 2018

**DOI:** 10.3201/eid2505.181534

**Published:** 2019-05

**Authors:** Yoon-Seok Chung, Jeong Min Kim, Heui Man Kim, Kye Ryeong Park, Anna Lee, Nam-Joo Lee, Mi-Seon Kim, Jun Sub Kim, Chi-Kyeong Kim, Jae In Lee, Chun Kang

**Affiliations:** Korea Centers for Disease Control and Prevention, Cheongju, South Korea (Y.-S. Chung, J.M. Kim, H.M. Kim, K.R. Park, A. Lee, N.-J. Lee, M.-S. Kim, J.S. Kim, C.-K. Kim, C. Kang);; Seoul Institute of Public Health and Environment, Seoul, South Korea (J.I. Lee)

**Keywords:** Middle East respiratory syndrome, coronavirus, Caco-2 cells, throat swab, sputum, *S* gene, spike gene, viruses, South Korea

## Abstract

We evaluated genetic variation in Middle East respiratory syndrome coronavirus (MERS-CoV) imported to South Korea in 2018 using specimens from a patient and isolates from infected Caco-2 cells. The MERS-CoV strain in this study was genetically similar to a strain isolated in Riyadh, Saudi Arabia, in 2017.

Between 2012 and the end of August 2018, a total of 2,248 laboratory-confirmed cases of Middle East respiratory syndrome coronavirus (MERS-CoV) infection and 798 associated deaths (case-fatality rate 35.3%) were reported in 27 countries (*1*). Most of these cases (83%) were reported in Saudi Arabia (1,871 cases, including 724 related deaths, for a case-fatality rate of 38.7%). 

The 2015 MERS-CoV outbreak in South Korea resulted in 186 laboratory-confirmed cases and 38 deaths. MERS-CoV spread via intrahospital and interhospital transmission at 16 clinics and hospitals across South Korea (*2*). This outbreak was recorded as the largest MERS outbreak outside of the Middle East (*3*). 

In August 2018, eight laboratory-confirmed cases of MERS-CoV infection were reported in Saudi Arabia, with 4 associated deaths; 1 case in a person who had travel history to the Arabian Peninsula was reported in the United Kingdom (*4*). In September 2018, a man in South Korea with a history of travel to the Middle East became ill and was suspected of having MERS-CoV infection. We report the investigation of this case and genetic characterization of the virus on the basis of variation and phylogenetic analyses of the spike (S) gene and the full-length genome from patient specimens and isolates from Caco-2 cells.

## The Study

On September 7, 2018, a 61-year-old man who had moved from Kuwait to South Korea visited the hospital with diarrhea and fever (*5*). The patient had a history of visiting the Middle East and was suspected of MERS-CoV infection; accordingly, he was transferred to a hospital with a national isolation ward.

On September 8, we collected nasopharyngeal swab and sputum samples from the patient and transported them to the Seoul Provincial Institute of Public Health and Environment (Seoul, South Korea). Real-time reverse transcription PCR showed that the sputum was positive for the upstream regions of the E protein (*upE*) and *orf1a* genes (*6*). Later the same day, we collected nasopharyngeal swab, throat swab, and sputum samples from the patient for further confirmation. The Korea Centers for Disease Control and Prevention (KCDC) confirmed that throat swabs and sputum samples were positive for *upE* and *orf1a*.

We added throat swab and sputum samples from the patient to a monolayer of Caco-2, Huh 7, and Vero E6 cells, which we incubated at 37°C in 5% CO_2_. We observed a cytopathic effect on the third day after inoculation in Caco-2 cells and confirmed viral replication of MERS-CoV in the supernatant of the infected cells by real-time reverse transcription PCR.

We extracted viral RNA from the specimens and the supernatant of the Caco-2 cells using the Viral RNA Mini Kit (QIAGEN, http://www.qiagen.com). We performed cDNA synthesis using the Superscript IV First-Strand synthesis system (Thermo Fisher Scientific, http://www.thermofisher.com) with random hexamers. We amplified cDNA by overlapping PCR to generate products of 600–1,100 bp covering the entire S gene and the full-length genome; we sequenced the resulting PCR amplicons by Sanger sequencing using an ABI 3730 Analyzer (Applied Biosystems, http://www.thermofisher.com).

**Figure 1:**
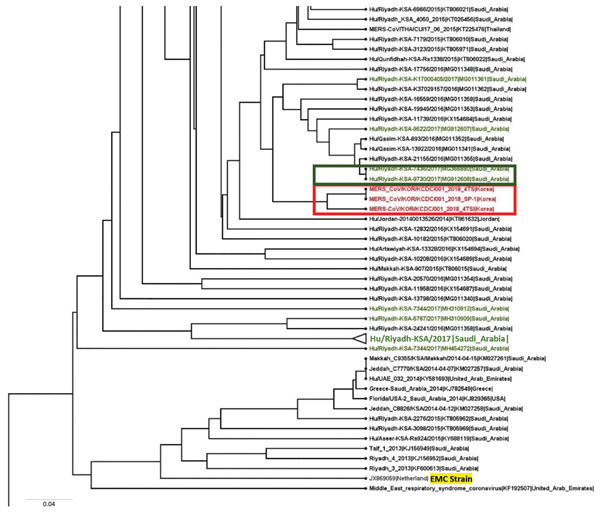
Molecular phylogenetic tree and coding region variants for the spike (S) glycoprotein gene of MERS-CoV isolates from South Korea, September 2018, and reference sequences. Phylogenetic analysis of 157 *S* sequences was performed using MEGA7 (https://www.megasoftware.net), with tree visualization using FigTree version 1.4.3 (http://tree.bio.ed.ac.uk/software/figtree). The taxonomic positions of circulating strains from the outbreak in South Korea and Riyadh, Saudi Arabia, are indicated. Boldface indicates compressed major clades of MERS-CoV. Bootstrap values (>70%) on nodes are shown as percentages based on 1,000 replicates. Red indicates South Korea 2018 isolates, blue indicates South Korea 2015 isolates, and green indicates Saudi Arabia 2017 isolates. The highlighted strain, from 2012, is the prototype strain used as the reference. EMC, Erasmus Medical Center; MERS-CoV, Middle East respiratory syndrome coronavirus. Scale bar indicates nucleotide substitutions per site.

**Figure 2:**
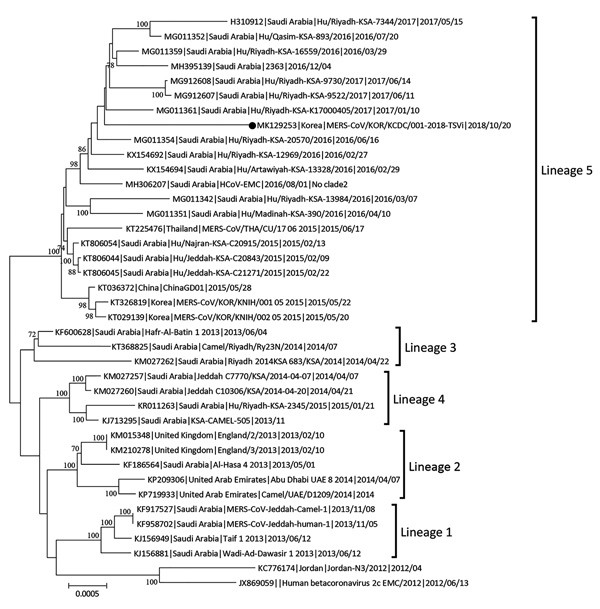
Maximum-likelihood tree showing MERS-CoV isolates from South Korea, September 2018 (black dot), and reference MERS-CoV genomes. Tree estimated using RAxML values (https://github.com/stamatak/standard-RAxML) on branches are shown as percentages based on 1,000 bootstrap replicates from the nucleotide sequences. MERS-CoV, Middle East respiratory syndrome coronavirus. Scale bar indicates nucleotide substitutions per site.

We used ClustalW (http://www.clustal.org) to align the S gene sequences of the isolates from the patient, MERS-CoV/KOR/KCDC/001_2018/SP-1 (from sputum sample), TS-1 (from throat swab sample), and TSVi (from Caco-2 processing), with those of 157 other MERS-CoV strains from GenBank and the MERS-CoV sequence database (https://www.ncbi.nlm.nih.gov/genomes/VirusVariation/Database/nph-select.cgi?cmd=database&taxid=1335626), deposited up to August 2018. We sequenced and analyzed the full-length genome of MERS-CoV/KOR/KCDC/001_2018/TSVi and 39 other MERS-CoV strains. We constructed phylogenetic trees by the maximum-likelihood method with 1,000 bootstrap replicates using MEGA7 (*7*) and RAxML (https://github.com/stamatak/standard-RAxML). We constructed phylogenetic trees from the MERS-CoV S genes (4,062 bp) (Figure 1) and the full-length genome (30,150 bp) of the virus (Figure 2) obtained from the patient in this study and the most similar human MERS-CoV sequences from other countries (*4*).

We submitted viral S gene sequences MERS-CoV/KOR/KCDC/001_2018_SP-1, TS-1, TSVi from the patient to GenBank under accession nos. MH978886, MH978887, and MH978888. MERS-CoV/KOR/KCDC/001_2018 SP-1, TS, and TSVi were most closely related (99.85%–99.90% nucleotide identity) to a human MERS-CoV strain isolated in Riyadh, Saudi Arabia, in 2017 (Hu-Riyadh-KSA-9730_2017; GenBank accession no. MG912608). KOR/KCDC/2018 SP-1 and TS had 5 nucleotide substitutions (C309T, T519C, C2928T, T3375C, and T3598C) compared with a MERS-CoV strain found in Riyadh in 2017, whereas MERS-CoV/KOR/KCDC/001_2018 TSVi had 3 nucleotide substitutions (C309T, C2928T, and T3375C) compared with the same strain. There were no substitutions in the N terminal domain (NTD) and receptor-binding domain (RBD) of the S gene in the same comparison (Table). We submitted the full-length genome sequences from MERS-CoV/KOR/KCDC/001_2018_TSVi, obtained from cell isolates, to GenBank under accession no. MK129253. In a phylogenetic analysis, the virus was located in a lineage of locally endemic 2016 and 2017 Saudi Arabia strains (lineage 5 in Figure 2) but was distinguished from 2015 South Korea strains.

**Table:** Comparison of spike glycoprotein gene sequence variants for a MERS-CoV isolates from a patient in South Korea, 2018, and reference strains*

MERS-CoV strain	Nucleotide (amino acid) positions										
NTD				RBD				Other regions		
183 (61)	258 (86)	409 (137)		1586 (529)	1588 (530)	1848 (616)		1886 (629)	3177 (1059)	3267 (1089)
JX869059_HCoV-EMC_2012†	C (G)	T (V)	A (S)		T (I)	G (V)	T (V)		G (R)	T (D)	C (S)
KR011266_KSA_Hu/Riyadh-KSA-2049/2015		C (V)									
KR011263_KSA_Hu/Riyadh-KSA-2345/2015		C (V)									
KR011264_KSA_Hu/Riyadh-KSA-2343/2015		C (V)									
KR011265_KSA_Hu/Riyadh-KSA-2466/2015		C (V)									
KT026453_KSA_Hu/Riyadh-KSA-2959/2015		C (V)									
KT026454_KSA_Hu/Riyadh_KSA_4050/2015		C (V)									
KX154690_Hu_Jeddah-KSA-161RS1146_2016		C (V)									
KX154684_Hu-Riyadh-KSA-11739_2016		C (V)									
KX154694_Hu-Artawiyah-KSA-13328_2016		C (V)									
MG011361_Hu-Riyadh-KSA-K17000405_2017		C (V)									
MG011360_Hu-Riyadh-KSA-K17000887_2017		C (V)									
**MG912608_Hu-Riyadh-KSA-9730_2017**		C (V)									
MERS-CoV/KOR/KNIH/001_05_2015		C (V)			C (T)					C (D)	T (S)
KT029139-MERS-CoV/KOR/KNIH/002_05_2015		C (V)	C (R)			C (L)				C (D)	T (S)
MERS-CoV/KOR/KNIH/009_05_2015		C (V)					C (V)			C (D)	T (S)
KT006149-ChinaGD01_2015	G (G)	C (V)					C (V)			C (D)	T (S)
MERS-CoV/KOR/KNIH/012_05_2015		C (V)			C (T)		C (V)			C (D)	T (S)
MERS-CoV/KOR/KNIH/013_05_2015		C (V)			C (T)		C (V)			C (D)	T (S)
MERS-CoV/KOR/KNIH/015_05_2015		C (V)			C (T)		C (V)		A (H)	C (D)	T (S)
MERS-CoV/KOR/KNIH/042_04_2015		C (V)								C (D)	T (S)
**MERS-CoV/KOR/KCDC/001_2018_2SP-1**		C (V)									
**MERS-CoV/KOR/KCDC/001_2018_4TS**		C (V)									
**MERS-CoV/KOR/KCDC/001_2018_4TSVi**		C (V)									

*Only variations from the reference strain are shown. Boldface indicates the South Korea isolates and the most similar strain identified in this study. MERS-CoV, Middle East respiratory syndrome coronavirus; NTD, N-terminal domain; RBD, receptor-binding domain.  †Reference MERS-CoV strain.

## Conclusions

We characterized the S gene and full-length genome of MERS-CoV imported to South Korea from the Middle East using sputum and throat swab specimens from a patient and from Caco-2 cells exposed to throat swab samples from the patient (*8**,**9*). A comparative genetic analysis of 157 strains isolated in 27 countries including South Korea demonstrated that the imported MERS-CoV we isolated showed the highest identity (99.85%–99.90%) with a strain recently isolated in Saudi Arabia in 2017 (*1**,**10*). These strains differed at 2 positions (C309T and T519C). We detected no variation in NTD or RBD, which are the major functional sites of the S protein (*11*). In addition, the strains involved in the outbreak in South Korea in 2015 had 12 nt substitutions (NTD, 1 site; RBD, 2 or 3 sites), implying that the imported case in 2018 involved a different virus (*4*).

Despite 110 laboratory-confirmed cases of MERS-CoV reported globally during 2018, no genetic information has been obtained to date. Therefore, we could not compare the genetic relationships between the strain we isolated and MERS-CoV strains in the Middle East (*12*).

In summary, we obtained genetic information about a MERS-CoV strain in South Korea in 2018 that was probably imported from the Middle East. We expect the sequence data we identified to support future epidemiologic investigations of MERS-CoV, particularly to fill the need for recent data. Furthermore, the results of this study improve our understanding of the evolution of MERS-CoV and highlight the need for enhanced surveillance, especially for persons traveling to the Middle East.

## References

[1] Corman VM, Ithete NL, Richards LR, Schoeman MC, Preiser W, Drosten C, et al. Rooting the phylogenetic tree of middle East respiratory syndrome coronavirus by characterization of a conspecific virus from an African bat. J Virol. 2014;88:11297–303. 10.1128/JVI.01498-14 25031349 PMC4178802

[2] Lee JY, Kim YJ, Chung EH, Kim DW, Jeong I, Kim Y, et al. The clinical and virological features of the first imported case causing MERS-CoV outbreak in South Korea, 2015. BMC Infect Dis. 2017;17:498–507. 10.1186/s12879-017-2576-5 28709419 PMC5512736

[3] Kim KH, Tandi TE, Choi JW, Moon JM, Kim MS. Middle East respiratory syndrome coronavirus (MERS-CoV) outbreak in South Korea, 2015: epidemiology, characteristics and public health implications. J Hosp Infect. 2017;95:207–13. 10.1016/j.jhin.2016.10.008 28153558 PMC7114867

[4] Plipat T, Buathong R, Wacharapluesadee S, Siriarayapon P, Pittayawonganon C, Sangsajja C, et al. Imported case of Middle East respiratory syndrome coronavirus (MERS-CoV) infection from Oman to Thailand, June 2015. Euro Surveill. 2017;22:30598. 10.2807/1560-7917.ES.2017.22.33.30598 28840828 PMC5572941

[5] World Health Organization. Middle East respiratory syndrome coronavirus (MERS-CoV) infection—Republic of Korea. 2018 Sep 12 [cited 2018 Dec 28]. http://www.who.int/csr/don/12-september-2018-mers-republic-of-korea/en

[6] World Health Organization. Laboratory testing for Middle East respiratory syndrome coronavirus: interim guidance (revised). 2018 Jan [cited 2018 Dec 28]. http://www.who.int/csr/disease/coronavirus_infections/mers-laboratory-testing/en

[7] Kumar S, Stecher G, Tamura K. MEGA7: Molecular Evolutionary Genetics Analysis version 7.0 for bigger datasets. Mol Biol Evol. 2016;33:1870–4. 10.1093/molbev/msw054 27004904 PMC8210823

[8] Muth D, Corman VM, Meyer B, Assiri A, Al-Masri M, Farah M, et al. Infectious Middle East respiratory syndrome coronavirus excretion and serotype variability based on live virus isolates from patients in Saudi Arabia. J Clin Microbiol. 2015;53:2951–5. 10.1128/JCM.01368-15 26157150 PMC4540943

[9] Drosten C, Muth D, Corman VM, Hussain R, Al Masri M, HajOmar W, et al. An observational, laboratory-based study of outbreaks of middle East respiratory syndrome coronavirus in Jeddah and Riyadh, kingdom of Saudi Arabia, 2014. Clin Infect Dis. 2015;60:369–77. 10.1093/cid/ciu812 25323704 PMC4303774

[10] Kossyvakis A, Tao Y, Lu X, Pogka V, Tsiodras S, Emmanouil M, et al. Laboratory investigation and phylogenetic analysis of an imported Middle East respiratory syndrome coronavirus case in Greece. PLoS One. 2015;10:e0125809. 10.1371/journal.pone.0125809 25919137 PMC4412533

[11] Kim DW, Kim YJ, Park SH, Yun MR, Yang JS, Kang HJ, et al. Variations in spike glycoprotein gene of MERS-CoV, South Korea, 2015. Emerg Infect Dis. 2016;22:100–4. 10.3201/eid2201.151055 26691200 PMC4696701

[12] Tsiodras S, Baka A, Mentis A, Iliopoulos D, Dedoukou X, Papamavrou G, et al. A case of imported Middle East Respiratory Syndrome coronavirus infection and public health response, Greece, April 2014. Euro Surveill. 2014;19:20782. 10.2807/1560-7917.ES2014.19.16.20782 24786258

